# Case report: Intraosseous hibernoma (IOH) mimics osseous metastasis: another rare pitfall in FDG-PET-CT

**DOI:** 10.3389/fnume.2023.1150143

**Published:** 2023-05-17

**Authors:** Sebastian J. Stolte, Hanna Geiger, Flavio Forrer, Regulo Rodriguez, Joachim Müller

**Affiliations:** ^1^ Department of Radiology and Nuclear Medicine, Cantonal Hospital St. Gallen, St. Gallen, Switzerland; ^2^ Department of Pathology, Cantonal Hospital St. Gallen, St. Gallen, Switzerland

**Keywords:** intraosseous hibernoma, hibernoma, FDG-PET/CT, osseous metastasis, adipocytic tumor, brown fat

## Abstract

Intraosseous hibernoma (IOH) mimicking osseous metastasis is a rare and little-known pitfall in nuclear medicine and radiology. Referring to a clinical case, we show imaging features in FDG-PET and CT as well as pathological characteristics and discuss MRI and differential diagnoses. A 73-year-old woman was assigned for an FDG-PET/CT examination after the incidental finding of a suspicious pulmonary nodule. The FDG-PET/CT examination detected a small slightly FDG-avid pulmonary nodule suspicious for malignancy and a small slightly sclerotic lesion with mild FDG-uptake in the upper pubic bone. Histopathology revealed an intraosseous hibernoma, a rare benign soft-tissue tumor arising from brown fat. In the sparse literature available, intraosseous hibernomas may or may not be positive on bone scans. As in our case, most are slightly sclerotic on CT but lytic lesions have also been described. On MRI, they are T1 hypointense to subcutaneous fat and hyperintense to skeletal muscle; they are usually T2 hyperintense and may show peripheral contrast enhancement. According to the literature, IOHs are mostly incidental findings with solitary lesions in the spine, pelvis, ribs, or, very rarely, in the extremities with low to moderately increased glucose metabolism. IOHs present as painless tumors in general; a few painful cases could be successfully treated with radiofrequency ablation or surgery. Differential diagnoses include metastases, lymphoma, fibrous dysplasia, and non-ossifying fibroma among others. Intraosseous hibernoma is a rare benign tumor that can mimic metastases in FDG-PET, CT, bone scan, and MRI. IOHs might be indistinguishable from metastases or malignant lesions, which makes a biopsy or follow-up mandatory in clinically relevant cases. Given the benign nature of IOHs, radiofrequency ablation or surgery is only an option in symptomatic cases.

## Introduction

It is well known that F-18-FDG (fluorodeoxyglucose) is far from being tumor-specific; nevertheless, FDG is the most commonly used PET tracer in nuclear medicine today. Many benign pathologies or anomalies in FDG-PET/CT can be, with some certainty, distinguished from metastases by their location, CT morphology, or distribution, such as sarcoidosis or activated brown adipose tissue. We present a rare pitfall in FDG-PET/CT that is not very well known and is difficult to distinguish from osseous metastases: intraosseous hibernoma (IOH), a rare benign tumor consisting of brown adipose tissue. In our case, the IOH appeared as a roundish sclerotic lesion with mild FDG-uptake, initially suspicious for metastasis. In the sparse literature available, intraosseous hibernomas may or may not be positive on bone scans. As in our case, most are slightly sclerotic on CT but lytic lesions have also been described (1). On MRI, they are T1 hypointense to subcutaneous fat and hyperintense to skeletal muscle; they are usually T2 hyperintense and may show peripheral contrast enhancement (2). On FDG-PET/CT, they have a low to moderately increased FDG-uptake (3). IOHs are mostly asymptomatic; nevertheless, radiofrequency ablation (RFA) and surgery are described in painful cases (2).

## Case report

A 73-year-old woman was assigned for an FDG-PET/CT examination after the incidental finding of a suspicious pulmonary nodule in a CT examination performed because of prolonged dyspnea in the context of an infection-related chronic obstructive pulmonary disease exacerbation without clinical improvement after 8 days of Prednisone therapy.

**Figure 1:**
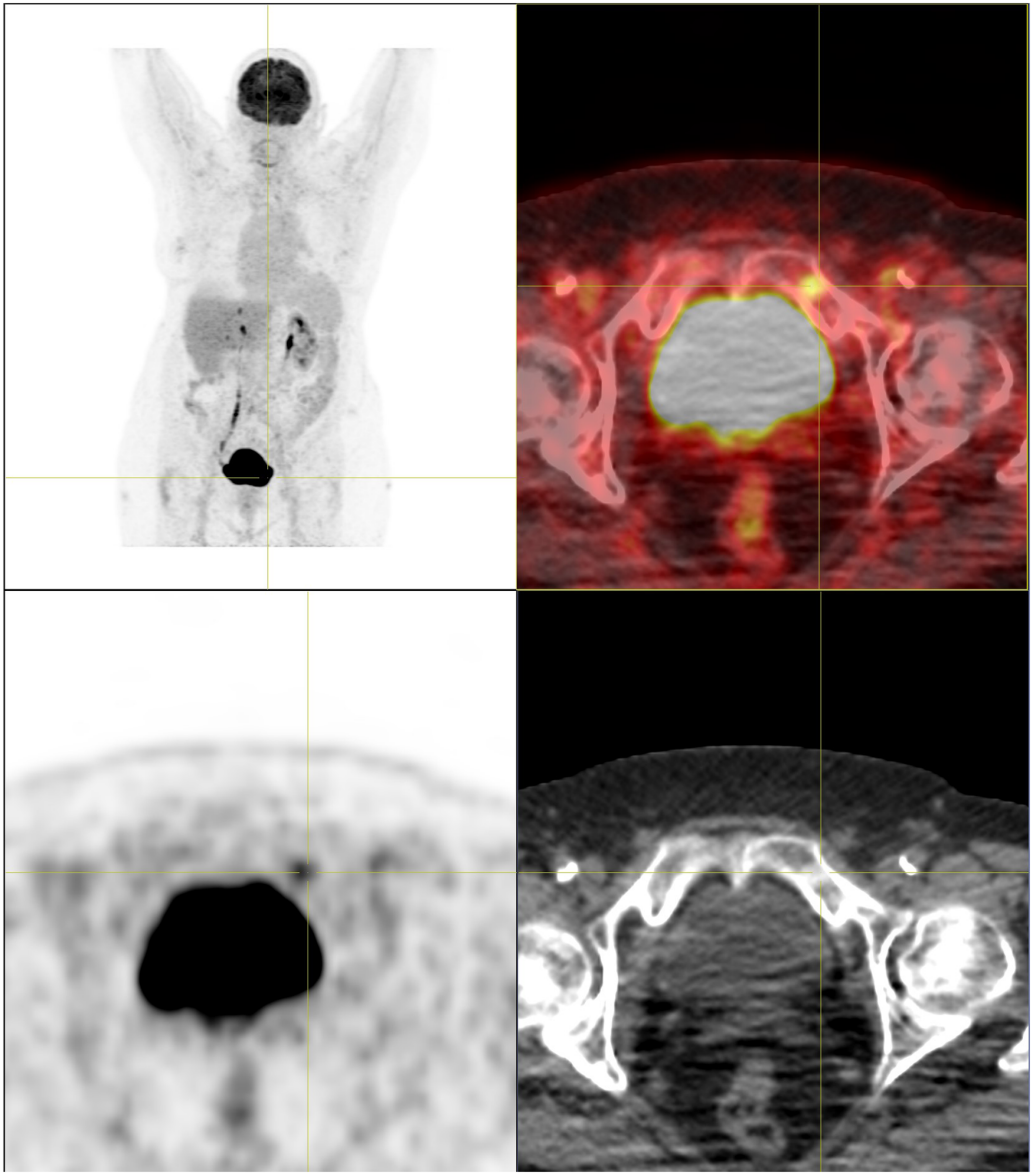
FDG-PET/CT. Roundish sclerosing lesion with low FDG-uptake in the upper left pubic branch.

**Figure 2:**
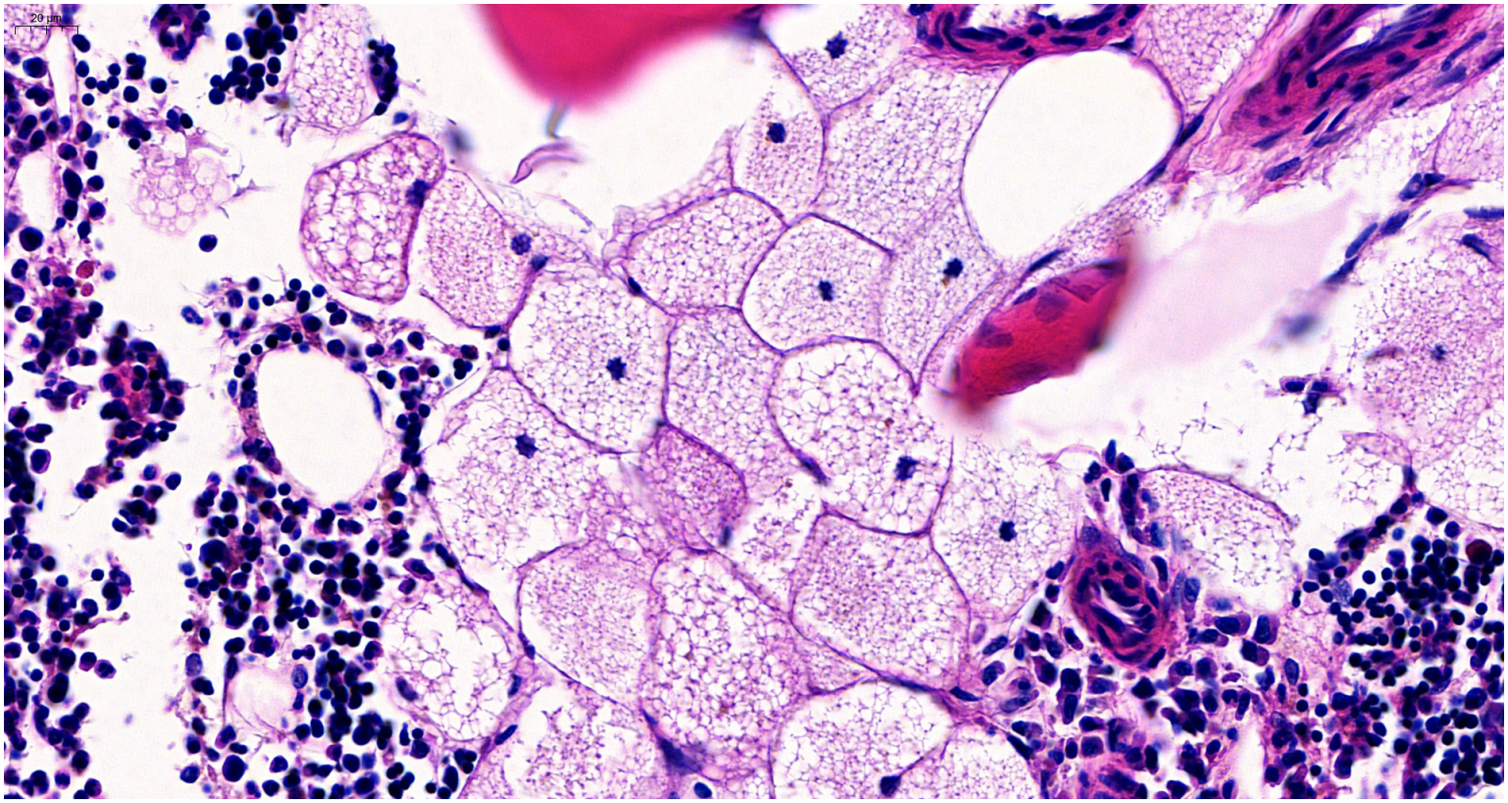
Histology. Histopathologic examination revealed brown fat cells in the bone marrow, solitary and with focal clustering. The cells typically show multiple small cytoplasmic vacuoles with a central, small nucleus slightly deformed by the homogeneous, small fat vacuoles. Bone remodeling or stromal changes could not be shown.

FDG-PET/CT revealed a small slightly FDG-avid pulmonary nodule in the upper lobe of the left lung with spiculated margins suspicious for malignancy, which later turned out to be a non-small-cell lung carcinoma. There was no evidence for FDG-avid or enlarged lymph nodes but a small, slightly sclerotic lesion with mild FDG-uptake (SUV_max_ 3.2) in the upper left pubic bone was detected (Figure 1). After finding adenocarcinoma metastases in a primarily unsuspicious infracarinal lymph node, the thoracic tumor board opted for a biopsy of the bone lesion. Histological work-up found regular hematopoietic bone marrow with an accumulation of moderately big, foamy cells with central small and inconspicuous nuclei (Figure 2). The possibility of foamy macrophages was ruled out in regard to their negativity for histiocyte markers CD11c and CD68.

The nodule was finally diagnosed as an IOH, a rare benign soft-tissue tumor composed of brown adipose tissue, which was indistinguishable from metastases during imaging.

## Discussion

The hibernoma is named after the brown adipose tissue that can be found in hibernating (winter-sleeping) animals (4). The brown adipose tissue is important for thermoregulation and is normally found in the head and neck region, paravertebral of the thoracic spine, and even in the region of the adrenal glands, predominantly in infants and young adults but can also be found in adults up to the ages of 40–50 years. When activated, as a response to the sympathetic activation *via* norepinephrine, the cells metabolize lipids and glucose, which consequently makes them avid for FDG (5).

The soft-tissue hibernoma is an uncommon but actually well-known tumor and is predominantly located in the subcutaneous and (less frequently) muscle tissue (6–8).

A hibernoma in general is a rare finding (9), first described in around 1906, with its function discovered later in 1914 (2). Intraosseous hibernoma, in contrast, only recently came to light in the literature. Hence, a Medline search revealed only 19 publications, nearly all of them being case reports, with 27 cases; a few more can be found in Google Scholar.

Lesion localizations are mostly in the spine and pelvis but also in the ribs and rarely in the extremities. Only one case report described two lesions in one patient; the other cases have been solitary lesions.

They are usually found incidentally during medical imaging for other reasons, mostly for the staging of carcinomas or orthopedic symptoms. That may be why the typical age of diagnosis is 40–85 years (10). IOHs are generally considered to be painless tumors, although they might occasionally present with pain. Surgery and radiofrequency ablation have been described only in three symptomatic patients.

Differential diagnoses include metastases, lymphoma, degenerative changes (e.g., sclerotic lesions at the iliosacral joint), healing fractures or bone contusions (e.g., of the ribs), fibrous dysplasia, non-ossifying fibroma, chordoma, liposarcoma, and more.

An IOH is a potential mimic of malignant lesions and can cause false-positive findings in FDG-PET scans. PET scans performed in seven reported cases showed variable metabolism, ranging from no hypermetabolism to mildly/moderately increased glucose avidity (SUV 2.5–4.6) (3). It has been hypothesized that this might be due to temperature-dependent activity of the adipocytes, with higher activity in cold temperatures and higher adrenaline levels (11).

An absolutely certain diagnosis is not possible by imaging alone. A follow-up or histological confirmation must be considered individually.

In histology, (intraosseous) hibernomas present as moderately big, foamy cells with unsuspicious nuclei and a high number of homogeneous, small lipid vacuoles, as they are found in physiological brown adipose tissue. Genetic analyses have identified some genetic alterations contributing to the development of hibernomas, such as an increase of UCP1 (3).

However, molecular criteria play a minor role in the diagnostic process as the histological morphology is fairly characteristic.

## Conclusion

An intraosseous hibernoma is a rare benign tumor that can mimic metastases during FDG-PET, CT, bone scan, and MRI.

Even though there are benign features in medical imaging, it might be indistinguishable from metastases or malignant lesions, especially since many IOHs are found incidentally during the work-up of malignancies. Consequently, a biopsy or follow-up examination must be taken into account to exclude malignancy depending on the situation of the individual patient.

Given the benign nature of IOHs, no treatment is necessary. RFA or surgery might be an option only in symptomatic cases (with localized pain).

## Data Availability

The raw data supporting the conclusions of this article will be made available by the authors, without undue reservation.
